# Free fibula flap for secondary mandibular reconstruction after gunshot injuries in Tigray, northern Ethiopia: A 5-year review of microsurgical challenges and outcomes in a war zone

**DOI:** 10.1007/s00508-025-02615-6

**Published:** 2025-09-19

**Authors:** Viktoria Koenig, Jakub Holoubek, Tomas Votruba, Julian Joestl, Tomas Kempny

**Affiliations:** 1 Department of Plastic, Aesthetic and Reconstructive Surgery, Medical University of Vienna, Waehringer Guertel 18–20, 1090 Vienna, Austria; 2 Department of Burns and Plastic Surgery, University Hospital Brno and Faculty of Medicine, Masaryk University, Brno, Czech Republic; 3 Department of Plastic Surgery, Hospital České Budějovice, Budějovice, Czech Republic; 4 Private Practice, Spitalgasse 19, 1090 Vienna, Austria; 5 Department of Plastic Surgery, MediCent Ostrava, President of Czech Society for Microsurgery, Ostrava, Czech Republic

**Keywords:** Microsurgery, Humanitarian mission, Mandibular reconstruction, Gunshot injuries, War surgery

## Abstract

**Background:**

Surgical care in Africa, particularly in conflict regions like Tigray, Ethiopia, faces unique challenges. Over 5 years a team of plastic and trauma surgeons conducted 5 humanitarian missions at Mekelle Referral University Hospital to reconstruct mandibular defects caused by shotgun injuries using free fibula flaps.

**Methods:**

This retrospective study analyzed 44 free fibula transfers performed between 2019 and 2023, with 33 cases involving mandibular reconstruction. Flaps were elevated using magnifying loupes; microscopes were employed when electricity was available. Postoperative flap monitoring and follow-up visits were conducted when feasible.

**Results:**

Of 33 mandibular reconstruction patients (3 females, 30 males; mean age 29.5 years), a microscope was available in only 28% of cases due to electricity interruptions. The mean surgery time was 532.7 min. The flap failure rate was 24% (8 of 33 cases), 8 microsurgical complications required intraoperative revision and 6 nonmicrosurgical complications (18%) were observed, primarily wound infections. Early outcomes varied: 21% had good results, 24% acceptable, 27% moderate, 24% no significant change and 3% worsened. Follow-up was incomplete; 29% of patients did not attend any postoperative visits.

**Conclusion:**

Microsurgical reconstruction in conflict-affected, resource-limited settings like Tigray is feasible but complicated by factors such as inconsistent electricity and loss to follow-up. Despite these challenges, acceptable complication and flap survival rates were achieved, highlighting the importance and feasibility of complex reconstructive surgery even under adverse conditions. Limitations include difficulties ensuring standardized operative environments and consistent long-term follow-up.

## Introduction

Vascularized bone grafting (VBG) is indispensable for reconstructing bony defects in maxillofacial injuries [1, 2]. Recently, there has been an increasing trend in mandibular gunshot injuries associated with the combat in the Tigray region of northern Ethiopia [3]. Projectiles used in combat and war zones often travel at high velocities, resulting in extensive destruction and avulsion of soft tissue and bone [4, 5]. Consequently, combat-related injuries therefore pose unique challenges to the restoration of form and function. The mandible is the most commonly injured structure in the maxillofacial complex during combat-related trauma [6]. It serves as a scaffold for crucial anatomical structures and plays vital roles in mastication, airway protection, articulation, and deglutition. The mandible also plays a significant role in facial esthetics, especially in the lower third of the face [7, 8]. There are various reconstruction options for mandibular defects including vascularized bone grafts (VBG), nonvascularized bone grafts (NVBG), alloplastic implants, reconstruction bars and distraction osteogenesis [9, 10]. Autogenous bone grafts, either vascularized or nonvascularized, are predominantly used in reconstructive surgery [5, 8, 11]. The aim of this study was the evaluation of the modalities and efficacy of mandibular reconstruction in combat-related injuries, particularly in resource-limited settings.

## Material and methods

### Patient identification and data collection

This study has been registered with Clinicaltrials.gov (ID: NCT06935916). The records included 44 patients who received free fibula transfers. The medical records of 33 patients who underwent free tissue transfers for mandibular reconstruction between 2019 and 2023 were reviewed in a retrospective setting analyzing the data and outcomes. Of the patients 11 had to be excluded due to incomplete data.

Clinical data were reviewed to identify patients’ demographics (age, mechanism of injury, side of injury, type of tissue transfer, average surgical time and complications).

Microsurgically related complications, including anastomosis complications, were assessed. Patient-related complications such as wound infections (superficial or deep), symptomatic pulmonary embolism, deep vein thrombosis (DVT), neurovascular injuries and postoperative death were recorded.

The management of patients was carried out in accordance with the guidelines of the local university hospital. All patients provided informed consent, with preoperative counselling conducted by local physicians, who also acted as translators when necessary. The tissue flaps were raised using surgical magnifying loupes. They were irrigated using a saline solution containing heparin, and a one-time injection of intravenous heparin was given during the surgery. All anastomoses were performed using microsurgical sutures and if available with a surgical microscope. Due to limited infrastructure and unreliable electricity, this was not possible in all cases.

Postoperatively, all patients were monitored in the intensive care unit for as long as required from an intensive care perspective. Flap monitoring was performed regularly by nursing staff under medical supervision. If indicated, a tracheostomy was performed prior to surgery. In instances where immediate reconstruction was undertaken, both the resection and reconstruction procedures were typically carried out by the same surgical team.

In this study microsurgical complications were defined as adverse events directly related to the microvascular anastomosis or flap viability, including partial or total flap loss, arterial or venous thrombosis, and need for urgent flap revision. Nonmicrosurgical complications encompassed all other perioperative or postoperative issues unrelated to the microvascular anastomosis, such as wound dehiscence, infection without flap compromise, or systemic complications.

Osteosynthesis was performed using standard 2.0 mm titanium miniplates and screws. Depending on the availability during each mission, fixation systems were sourced from established manufacturers such as DePuy Synthes (DePuy Synthes, West Chester, PA, USA) or Gebrüder Martin/KLS Martin Group (KLS Martin Group, Tuttlingen, Germany) or provided through humanitarian aid programs using surgical-grade equivalents. Plate contouring and fixation were carried out with the aim of adhering to international maxillofacial standards; however, due to limited on-site resources, full compliance with these standards was not always feasible. Intraoperative imaging with a C-arm was not consistently available, as image intensifiers were often nonfunctional or entirely absent. Consequently, implant positioning had to be assessed manually and visually, which posed an additional challenge under the prevailing conditions.

### Statistics

Statistical analysis was undertaken using R Version 3.1.1 SPSS (IBM, Armonk, NY, USA). Descriptive data (mean, median, range, proportions) are reported for the entire patient cohort. Differences between means and proportions were tested with the χ^2^-test for categorical variables and the unpaired t‑test for continuous variables. A probability value of *p* ≤ 0.05 is considered statistically significant.

## Results

Overall, 33 patients underwent free tissue transfer for mandibular reconstruction following shotgun injuries and 3 female and 30 male patients have been treated.

**Fig. 1:**
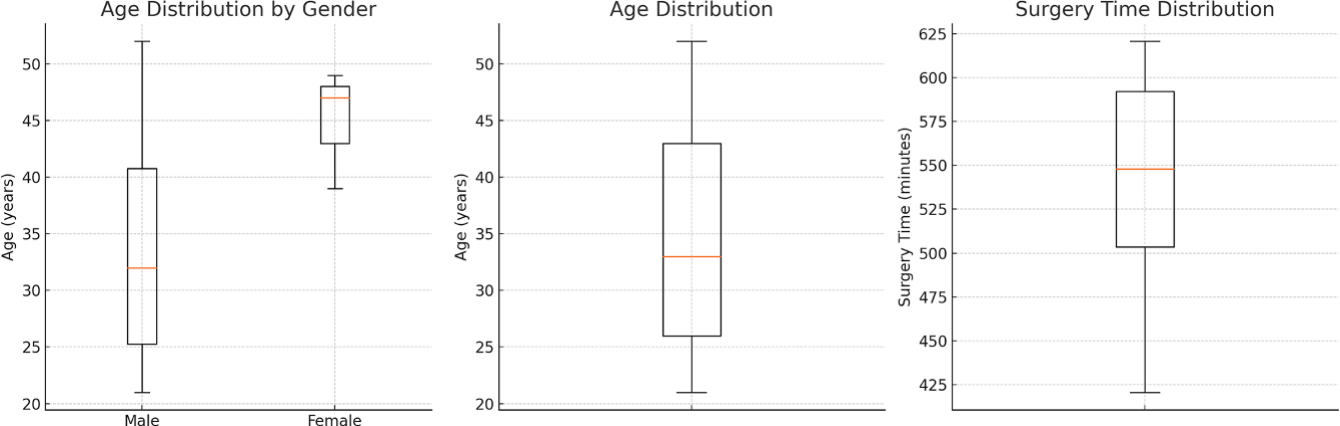
Demographic data and time of surgery

The mean age was 29.5 years at the time of the surgery with a range from 18–53 years. Mean surgery time was 532.7 min for maxillofacial patients (Fig. 1).

In all cases, the lead surgeon was the visiting specialist.

In 28% of the cases, standardized microsurgery techniques were utilized facilitated by a microscope at all times; however, it is important to note that for the remaining patients, these advanced techniques were not consistently feasible due to the unavailability of electricity. In such situations, surgery was continued using a flashlight and either a microscope or surgical loupes, although the conditions did not meet the standardized environments typically available in high-resource settings. Patients were followed up in the outpatient clinic for a median of 9 months, with a range from 3 weeks to 41 months. Currently, 35% of the patients are still under follow-up care; however, 29% of the patients did not attend any follow-up outpatient appointments after being discharged.

A total of 8 microsurgical complications with the anastomosis could be found, needing an intraoperative revision of either the venous or arterial anastomosis due to poor flap perfusion or drainage.

Nonmicrosurgical complications were found in 6 patients, which is 18%. The primary complication observed was wound infection in 5 cases, with an additional noteworthy complication encompassed bleeding in 1 case.

A total of 8 free fibula graft failures were observed in our mandibula reconstruction cases, which is approximately 24%. No osteosynthesis-related complications, such as plate loosening, malposition, or infection, were observed during the documented follow-up.

**Fig. 2:**
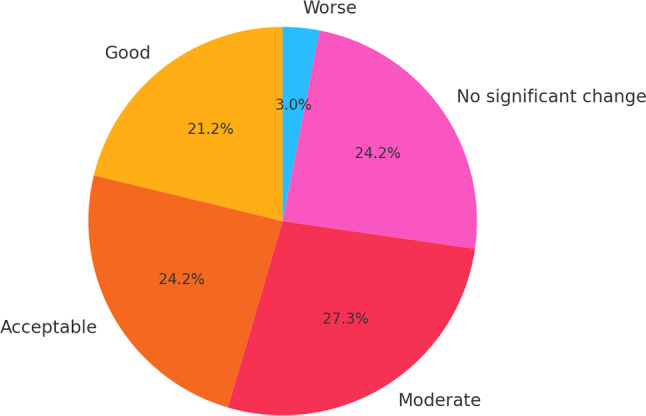
Pie chart showing the distribution of early clinical outcomes

**Fig. 3:**
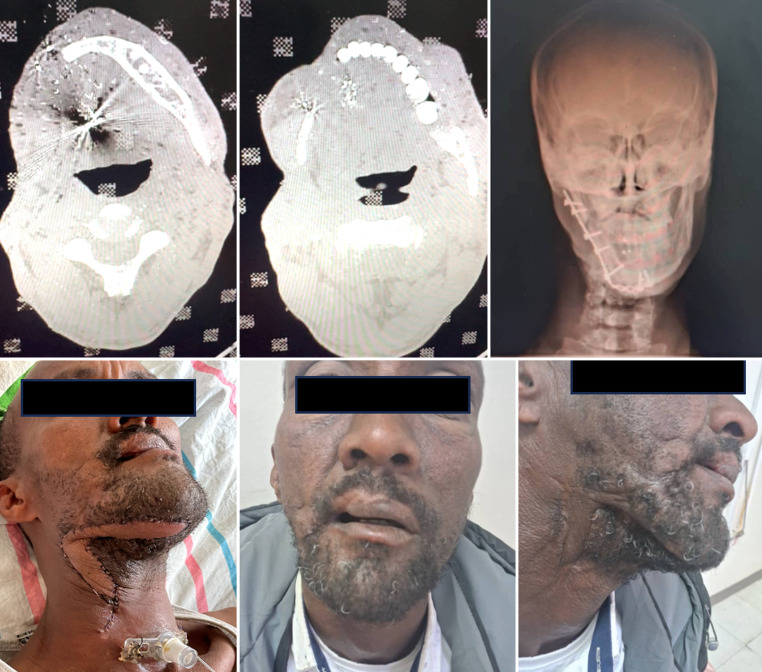
Case 1: secondary mandibula reconstruction after gunshot injury. **a**, **b** Preoperative CT scan of the mandible, showing the segmental defect after shotgun injury. **c** Postoperative X-ray confirming fixation of the free fibula flap with reconstruction plate (a postoperative CT was not performed due to limited availability and high costs for patients in Africa). **d**-**f** Postoperative clinical photograph, showing restored mandibular contour and demonstrating soft-tissue healing

**Fig. 4:**
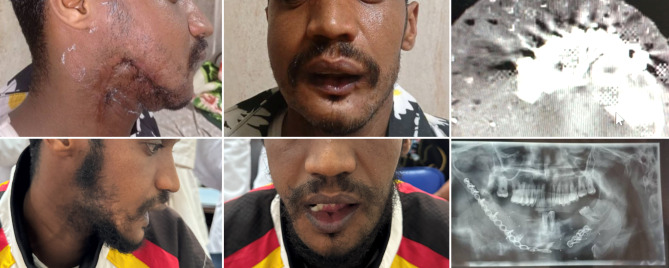
Case 2: secondary mandibula reconstruction after gunshot injury. **a**, **b** Preoperative clinical photograph. **c** Preoperative CT scan of the mandible demonstrating the segmental defect. **d**, **e** Postoperative clinical photograph. **f** Postoperative X-ray confirming fixation of the fibula flap with reconstruction plate and restoration of mandibular continuity

The early clinical outcome score for patients undergoing free fibula reconstruction revealed the following distribution: 7 patients achieved good results, 8 had acceptable outcomes, 9 experienced moderate outcomes, 8 reported no significant change and 1 patient experienced a worse outcome. When considering the total outcomes across all categories, the distribution is as follows: 21% of vascular fibula transfers performed achieved good results, 24% had acceptable outcomes, 27% showed moderate results, 24% had no significant change, and 3% ended up with a worse result (Figs. 2, 3 and 4).

## Discussion

Free fibula transfer has become an essential technique in mandibular reconstruction, offering reliable solutions for defects caused by trauma, tumors, infections, and congenital anomalies [11–13]. In high-income countries, it is considered the gold standard due to its proven effectiveness in restoring both function and esthetics [13]. Although both vascularized and nonvascularized bone grafts are used to reconstruct mandibular defects, the choice often depends on the setting and resources [6]. Nonvascularized bone grafts (NVBG), although simpler and more accessible, are generally less durable [8]. In contrast, vascularized bone grafts (VBG), particularly the free fibula flap, offer superior outcomes but are more technically demanding and associated with higher complication rates, especially in resource-constrained environments [7, 8]. Despite these inherent challenges, the advantages of the vascularized fibula flap are widely acknowledged [14]. Its long, dense segment of cortical bone is resistant to pressure and torsion and can accommodate osseointegrated implants. Moreover, its capacity for multiple osteotomies and soft tissue integration makes it ideal for reconstructing complex mandibular defects.

The success of such procedures hinges on several critical factors: robust vascularization to ensure graft survival, an accurate anatomical fit for functional and esthetic restoration, and meticulous surgical technique, often supported by preoperative virtual planning and 3D modeling [9, 10]. These tools, however, remain unavailable in much of sub-Saharan Africa due to cost and access limitations [15, 16].

Our own experience with mandibular reconstruction in the conflict-affected Tigray region must be viewed against this backdrop of global practice. Su et al. emphasize why the fibula flap continues to be the workhorse of mandibular reconstruction: it combines long vascularized bone, reliable soft tissue components, and compatibility with implant-based rehabilitation [14]; however, their description of ideal conditions, such as preoperative imaging, virtual surgical planning, and multidisciplinary teamwork, highlights the disparity in our context, where such resources are unavailable. Nonetheless, we observed that acceptable clinical outcomes can be achieved even under austere conditions, affirming the resilience and adaptability of the FFF.

In this context, Wang et al. underscore the value of digital planning in improving the precision of reconstruction and reducing surgical time [9]. Yet, our setting necessitates reliance on experience and adaptability rather than technology. Similarly, Kadam et al. point out the difficulties inherent in secondary reconstruction, particularly the management of fibrosis and distorted anatomy, which we frequently encountered and addressed through flexible, case-by-case strategies [7].

Rehman et al.’s findings further contextualize our outcomes [8]. Their study reports an 88.2% success rate using nonvascularized iliac bone grafts for small mandibular defects (4–7 cm), surpassing the success rate we observed with vascularized fibular grafts. This discrepancy may reflect the higher complexity of our cases, which aimed at restoring complete mandibular continuity and function under difficult conditions, often in patients with large, irregular defects and compromised soft tissue.

Further supporting our experience, Banda et al. conducted a meta-analysis of free flap surgeries in Africa, reporting a pooled flap survival rate of 89%, albeit with a relatively high complication rate of 51% [15, 17]. Our 24% graft loss and corresponding complication rate mirror these findings and illustrate the importance of perioperative protocols even in makeshift conditions. Our attempts at standardized monitoring and follow-up were critical in mitigating risks, although not always possible due to patient mobility and infrastructure limitations.

Walia et al. highlighted the feasibility and effectiveness of second free flaps after initial flap loss, with a reported success rate of 93% and lower complication rates than alternative salvage methods [18]. In our context, however, second free flaps are rarely feasible due to time, equipment, and staffing constraints. Instead, we focused on intraoperative revisions when perfusion issues were identified early.

In mandibular reconstruction, a novel approach is the “jaw in a day” concept, involving dental implant placement into the fibula during flap transfer under the same anesthesia as bone harvesting. A provisional prosthesis is promptly fitted to the neomandible, significantly expediting oral feeding resumption for the patient. While such approaches or other innovative practices like 3D printing for mandibula reconstruction are standard in high-resource settings, humanitarian missions must rely on basic techniques and aim to establish a treatment standard that is both feasible within the local infrastructure and available resources, and, above all, safe for the patient [15].

The value of structured, short-term collaborative missions is further emphasized by De Berker et al., who outlined the dual benefit of providing care and building capacity through training [19]. Our own missions followed a similar model, combining surgical intervention with infrastructure assessment, logistical planning, and local mentorship. What makes our contribution particularly significant is the inclusion of war-injured patients, a population rarely featured in published datasets from short-term reconstructive missions.

A valuable complementary perspective comes from Gebremariyam et al., who documented the creation of a microsurgical service in Jimma, Ethiopia [20]. Their emphasis on sustainable training, equipment procurement, and long-term planning mirrors our own aspirations; however, the active conflict and systemic instability in Tigray present ongoing challenges to similar capacity-building efforts in the region [3, 4]. Nevertheless, our repeated missions and preoperative evaluations helped prepare the local setting for complex microsurgical procedures and may serve as a foundation for future development once the situation stabilizes.

Surgical duration can vary significantly and is influenced by local resources as well as factors that are uncommon in high-income settings, such as non-functioning or unreliable electricity. While complex procedures are generally performed using a dual team approach, this method is also applied in the local context; however, efficiency is not always comparable as the local team is still in the process of developing microsurgical expertise. In their study on the challenges of global microsurgery, Citron et al. reported that the flaps which took the longest to perform were the fibula flaps, with a median of 508 min (interquartile range, IQR 453–558 min), due to the involved nature of fibula shaping for mandibular reconstruction [5]. This is roughly in line with our experience, where the mean surgery time for maxillofacial patients was 532 min.

The delivery of high-quality surgical care in such settings inevitably faces significant logistical and ethical challenges. Frequent power outages necessitate the use of headlights during surgery, and the absence of bipolar cautery and running water compels us to adapt traditional surgical techniques and sterilization methods. We also transport essential surgical equipment, including microsurgical instruments, battery-powered drills, and suture material, to ensure autonomy during operations. Customs and transport logistics, although legally supported, add a further layer of complexity.

Ethical decision-making plays a crucial role. We must continuously weigh the risks and benefits of major surgeries in patients who may not have consistent access to follow-up care. This is why we conduct premission site visits and engage closely with the local team to assess not only technical feasibility but also the sustainability of postoperative management. Fortunately, the intensive care unit (ICU) in Mekelle University Hospital is well-equipped by regional standards, which justified our decision to proceed with complex reconstructions. Preoperative diagnostic imaging was covered by our team, including necessary radiographs or CT scans prior to surgery. In several cases, we were also able to finance selected postoperative imaging; however, the extent of postoperative diagnostics did not match the standards typically expected in high-resource settings, due to both financial and logistical limitations.

Postoperative care continues through active collaboration with local medical teams, supported by remote communication with our team. Complications that arise are either addressed on-site or discussed in joint consultation, ensuring continuity of care despite the geographical and infrastructural hurdles.

Our findings align closely with those reported by Bouaoud et al., who analyzed 5 years of humanitarian maxillofacial missions in Senegal [21]. Similar to our experience, their missions predominantly addressed tumors and posttraumatic or postinfectious sequelae, with free flap reconstruction playing a central role in surgical management. Despite working in a resource-limited setting, they achieved low complication rates and favorable functional outcomes, emphasizing the value of meticulous planning, structured follow-up, and close collaboration with local teams. These parallels reinforce the importance of context-sensitive surgical strategies and highlight that with proper preparation microsurgical reconstruction can be safely and effectively implemented even under challenging conditions. Their emphasis on sustainable mission planning, ethical awareness, and capacity-building also echoes our own operational principles in Tigray.

The philosophy of global surgery increasingly emphasizes building local capacity rather than only providing care, and in principle, teaching local surgeons complex procedures such as microsurgical mandibular reconstruction with free fibular flaps is a realistic and important goal for sustainable surgical improvement; however, feasibility depends heavily on local factors like existing surgical experience, availability of essential equipment (e.g., microscopes, microinstruments), reliable infrastructure (consistent electricity, sterile environments), and perioperative support. In resource-limited or conflict-affected settings, even highly motivated surgeons may require 3–5 years of repeated hands-on training missions with close mentorship and gradual skill development before independently performing microsurgical reconstructions safely. Without stable infrastructure and team capacity, the learning curve is significantly extended, and outcomes risk being unacceptably poor. Therefore, while microsurgical training should remain a long-term objective, initial education should focus on foundational reconstructive techniques such as pedicled flaps and skin grafts, with progressive steps toward microsurgery as local systems, experience, and resources allow, supported by sustained collaboration and investment in local capacity.

## Conclusion

Mandibular reconstruction in Tigray faces challenges due to limited resources and a steep learning curve. Access to high-quality microsurgical instruments and sutures is constrained by cost and distribution issues. Despite early challenges, strategic procurement of quality materials and improved surgical expertise enhanced outcomes. Combat-related defects often use nonvascularized bone grafts (NVBGs) for their practicality, despite shaping challenges. Vascularized bone grafts (VBGs) like free fibula grafts offer superior functional and esthetic results due to better blood supply and integration. Enhancing surgical care, investing in essential equipment, and continuous training are essential for successful mandibular reconstruction in developing regions.

In summary, while our outcomes may fall short of those achieved in high-income settings or even well-supported low-income and middle-income countries (LMIC) centers, they demonstrate that complex microsurgical reconstruction, particularly mandibular free fibula transfer, is achievable and impactful in a war zone. This underscores the importance of tailored, context-sensitive strategies and the need for further capacity-building initiatives in post-conflict recovery.

### Limitations

One limitation of this study is the incomplete long-term follow-up: 29% of patients did not return for planned long term outpatient visits after discharge. This limited the availability of comprehensive long-term outcome data and may have introduced some uncertainty into the interpretation of late complications and overall success rates. Nonetheless, given the challenging conditions in the conflict-affected region and the inherent difficulties of patient tracking in such settings, the achieved follow-up rate is within what can reasonably be expected.
